# Supplementary material for: Room-temperature cavity exciton-polariton condensation in perovskite quantum dots
Source: Nat Commun. 2025 Jun 5;16:5228. doi: 10.1038/s41467-025-60553-3 (PMC12141476; doi:10.1038/s41467-025-60553-3)
Supplement: Supplementary file 1 — Supplementary Information [file 41467_2025_60553_MOESM1_ESM.pdf]

Supplementary Information: Room-temperature cavity exciton-polariton condensation in perovskite quantum dots

Ioannis Georgakilas[1,2,+], David Tiede[3,+], Darius Urbonas[1], Rafał Mirek[1], Clara Bujalance[3], Laura Caliò[3], Virginia Oddi[1,4], Rui Tao[4,5], Dmitry N. Dirin[4,5], Gabriele Rainò[4,5], Simon C. Boehme[4,5], Juan F. Galisteo-López[3], Rainer F. Mahrt[1], Maksym V. Kovalenko[4,5,*], Hernán Miguez[3,*], Thilo Stöferle[1,*]

[+]contributed equally

[*]corresponding authors: mvkovalenko@ethz.ch, h.miguez@csic.es, tof@zurich.ibm.com

[1]IBM Research Europe - Zurich, Säumerstrasse 4, 8803 Rüschlikon, Switzerland

[2]Institute of Quantum Electronics, Department of Physics, ETH Zürich, Auguste-Piccard-Hof 1, 8093 Zürich, Switzerland

[3]Multifunctional Optical Materials Group, Institute of Materials Science of Seville, Consejo Superior de Investigaciones Científicas - Universidad de Sevilla (CSIC-US), Américo Vespucio 49, Sevilla 41092, Spain

[4]Laboratory of Inorganic Chemistry, Department of Chemistry and Applied Biosciences, ETH Zürich, Vladimir-Prelog-Weg 1-5/10, 8093 Zürich, Switzerland

[5]Laboratory for Thin Films and Photovoltaics, Empa - Swiss Federal Laboratories for Materials Science and Technology, Ueberlandstrasse 129, 8600 Dübendorf, Switzerland

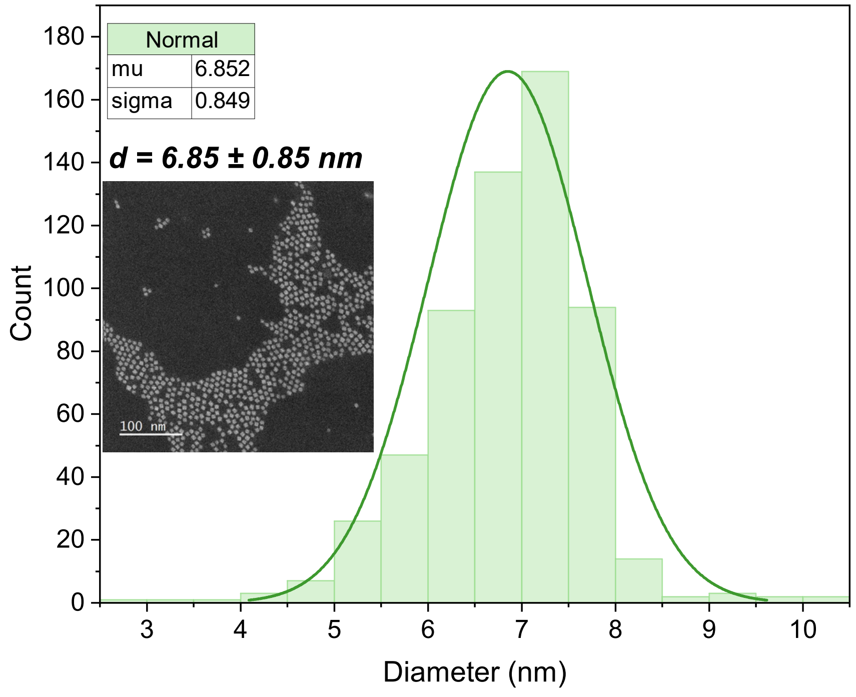

Supplementary Figure 1. **QD size distribution and scanning transmission electron microscopy (STEM).** The histogram shows the nanocrystal size distribution as obtained from image analysis (inset: STEM image). The solid line corresponds to a fitted normal distribution.

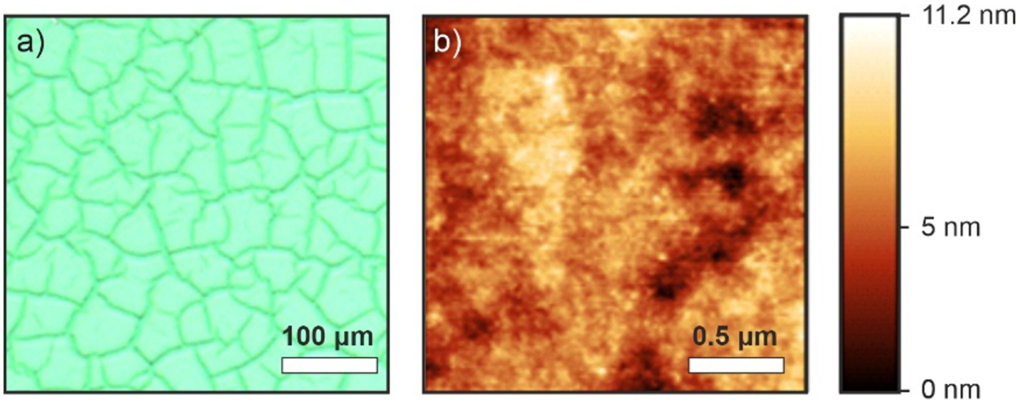

Supplementary Figure 2. **Surface morphology of perovskite QD solid thin film. a,** Optical imaging of the surface structure of a perovskite QD thin film deposited on the DBR substrate. Surface tension is released on "cracks" throughout the sample. Low surface roughness domains are formed between the cracks. **b,** Atomic-force microscopy (AFM) image of an area between cracks, showing a surface roughness of 1 - 2 nm rms.

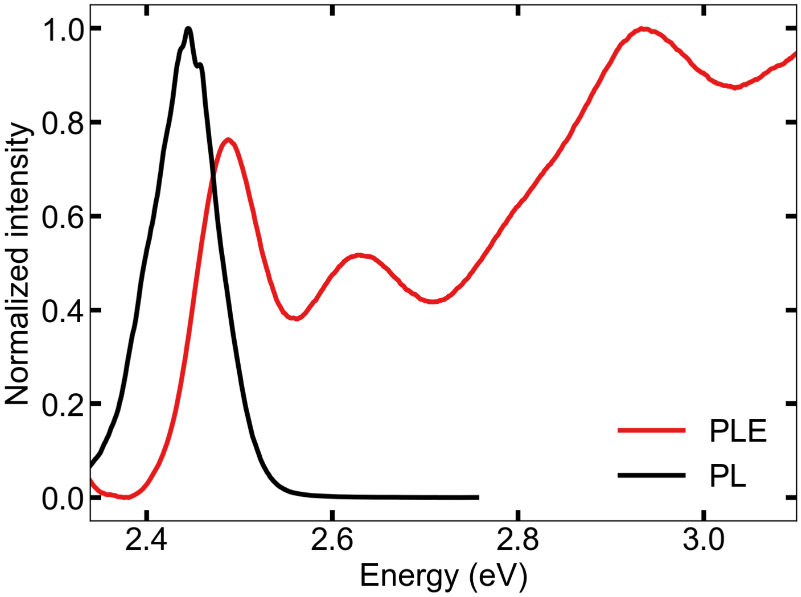

Supplementary Figure 3. **Optical properties of the perovskite QD solid thin film.** Normalized photoluminescence (black, PL) and photoluminescence excitation (red, PLE) spectrum of the QD solid film deposited on the DBR mirror, but without the other cavity half on top.

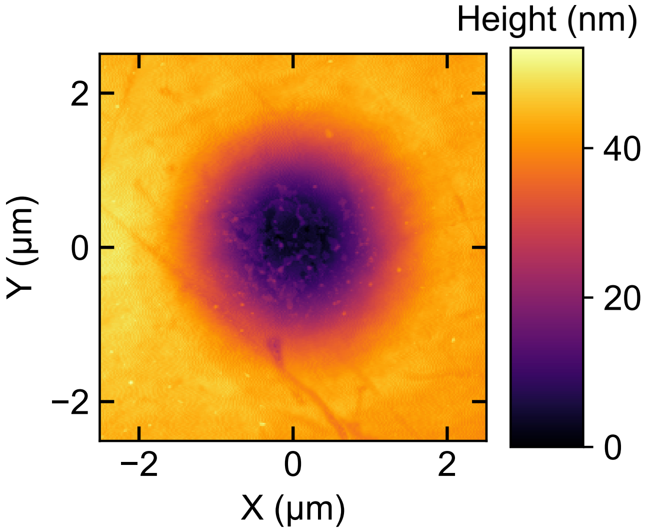

Supplementary Figure 4. **Atomic force microscopy image of the Gaussian-shaped deformation.** The Gaussian deformation fabricated with focused ion beam milling for this experiment has a FWHM of 2 μm and 45 nm depth and low surface roughness.

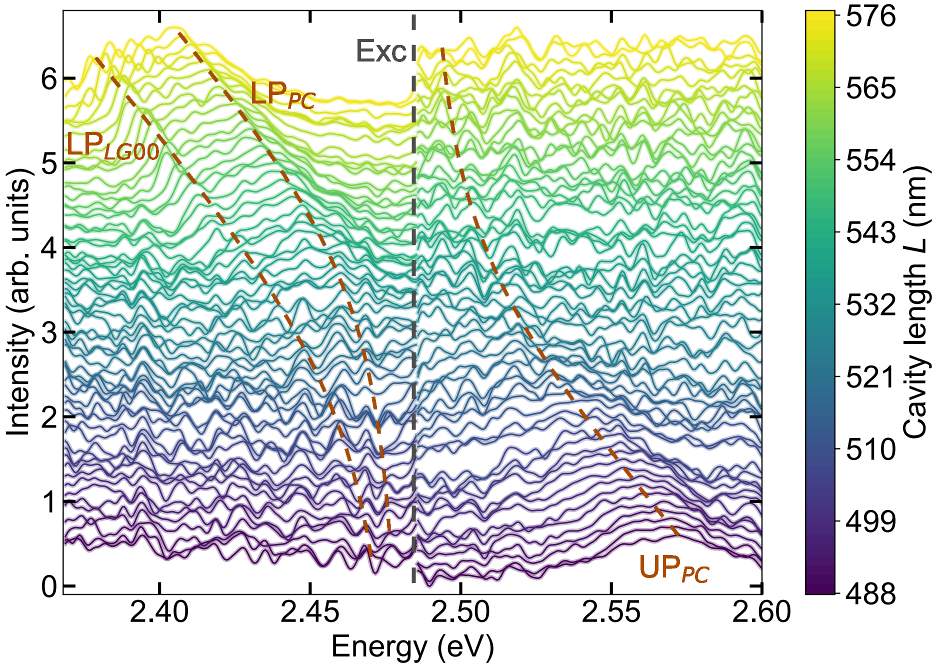

Supplementary Figure 5. **Waterfall plot of the measured white-light transmission spectra as a function of cavity length.** Measured transmission spectra belonging to different cavity lengths plotted with vertical spacing and different color for each spectrum. The red dashed lines indicate the fitted polariton dispersions for the LG00 and PC modes. The grey dashed line shows the energy position of the excitonic peak. The data to the left and right of this line are separately normalized to improve visibility.

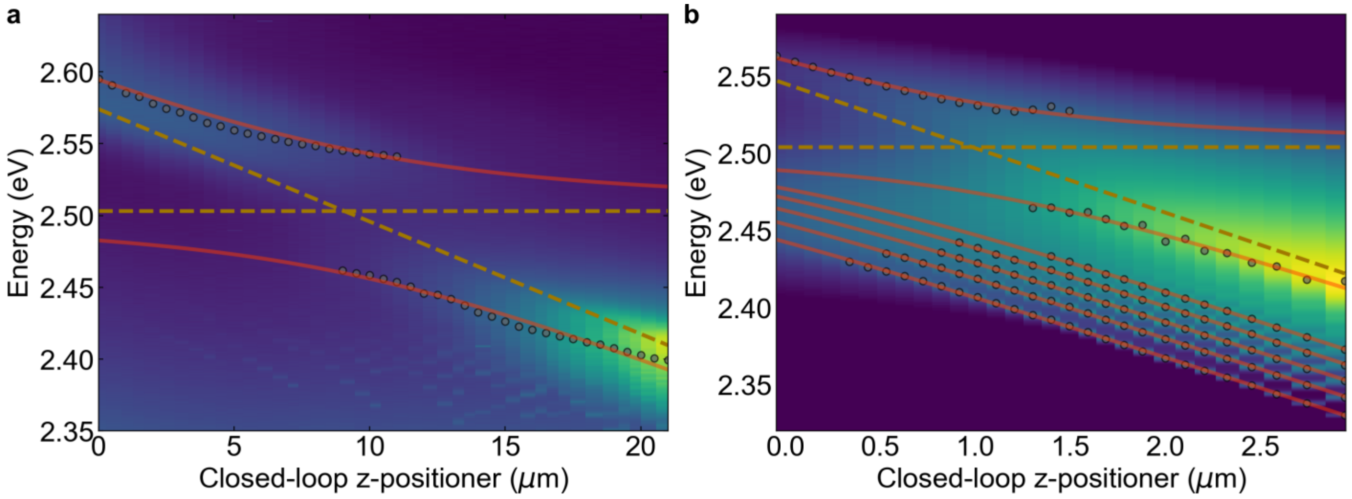

Supplementary Figure 6. **Additional white light transmission measurements demonstrating strong coupling. a,** Transmission measurement taken at a different position of the main sample as the one used in the manuscript, where we see the anticrossing of the PC mode with a fitted Rabi splitting of (87 ± 2) meV. **b,** Transmission measurement on a second, similar sample utilizing the same CsPbBr3 QDs. Here, we can observe the level tuning for both PC and LG modes. The fitted Rabi splitting for the PC mode is (59 ± 2) meV. In both panels the fits to the polariton dispersion are represented by the red solid lines, while the exciton and photon energies are given by the dashed orange lines. The extracted positions of the several peaks are indicated with the black circles. The raw closed-loop z-stage positions are shown as horizontal axes, and the marked difference between the samples is a result of different pressing / lever arm conditions when the top and bottom substrates are in contact.

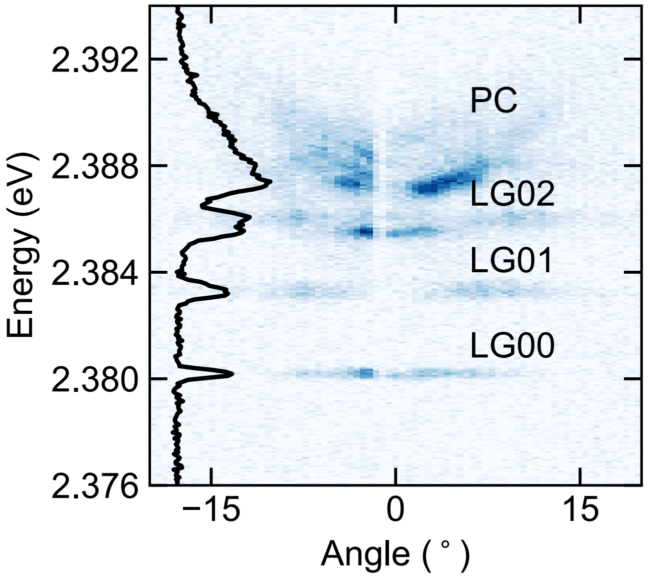

Supplementary Figure 7. **Cavity dispersion for identifying the various photonic modes.** The angular dispersion measured at large detuning from the exciton reveals the set of Laguerre-Gaussian modes originating from the Gaussian-shaped deformation and the planar cavity mode. As shown in the image, the lowest energy dispersionless mode corresponds to the LG00 mode while the highest energy parabolic mode corresponds to the planar cavity mode PC. On the left side of the panel, the respective angle-integrated spectrum (black solid line) is presented.

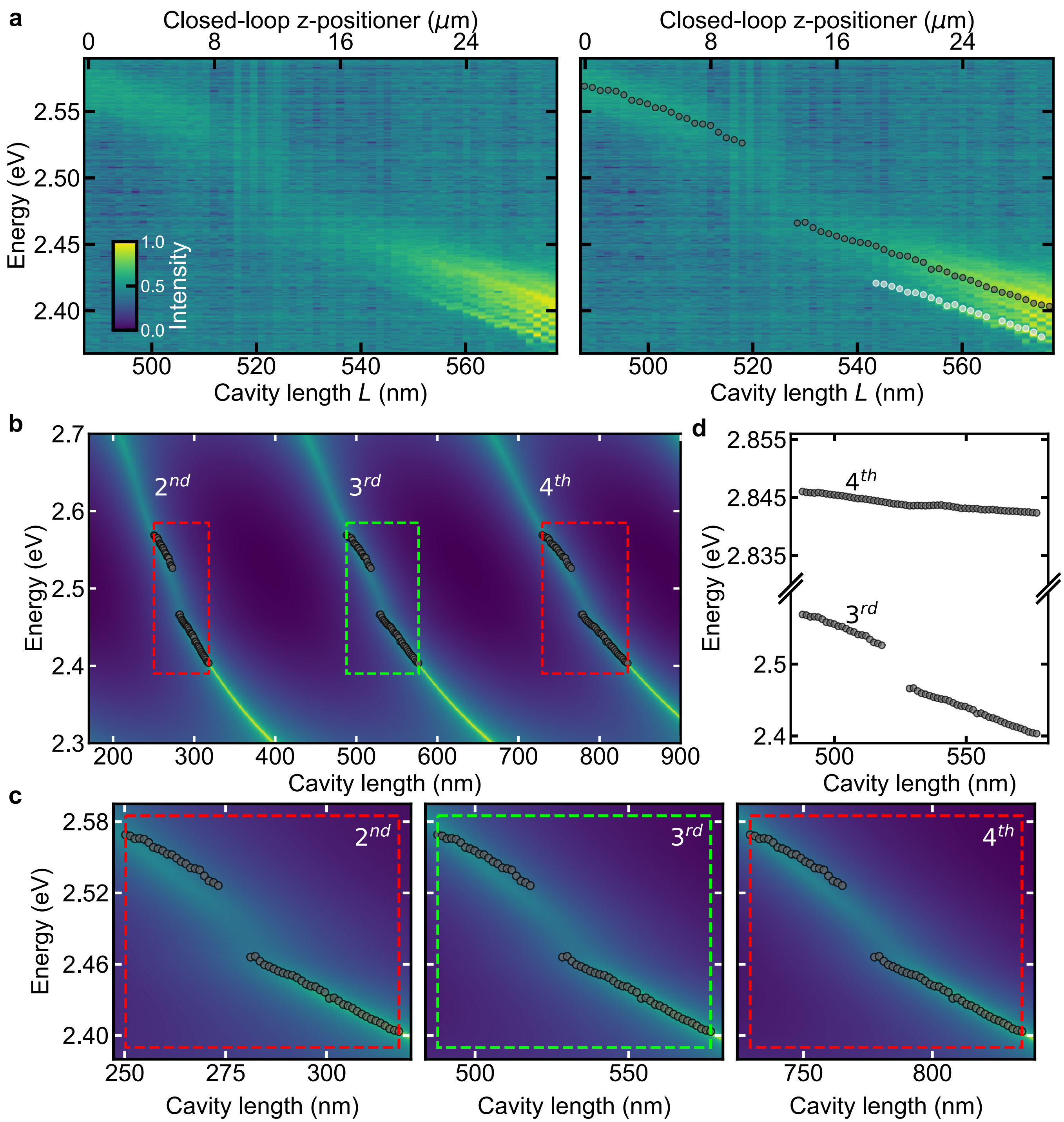

Supplementary Figure 8. **Transfer matrix simulation for cavity length calibration.** **a,** The left panel is the same measurement present in Fig. 1b but without the fits. The right panel is again the same measurement but additionally the extracted peak positions are highlighted with black circles for the PC mode and white circles for the LG00 mode. The top axis shows the raw closed-loop z-positioner value, which is translated through a lever-like motion into a much smaller change of cavity length. **b,** Transfer-matrix simulation of transmission spectra for a wide range of cavity lengths which includes the 2nd, 3rd and 4th longitudinal order cavity modes. By comparing the extracted data from the experimental polariton dispersion of the PC mode shown in (a) (black circles) to the simulation, we conclude that the experimental data match slightly better the region highlighted with the green dashed box compared to the red dashed box regions, therefore determining the length L of our cavity. **c,** Zoomed-in images of the areas inside the red and green dashed boxes from panel (b). Here, it becomes even more apparent that the overlap between the extracted experimental data and the simulated dispersion inside the green box area pair the best. **d,** Extracted experimental data from the transmission measurement, showing both the 3rd and 4th longitudinal order PC modes. The 4th order mode, which is far from the exciton, tunes linearly which indicates linear pressing between the cavity halves and true linear, lever-like modification of the cavity length.

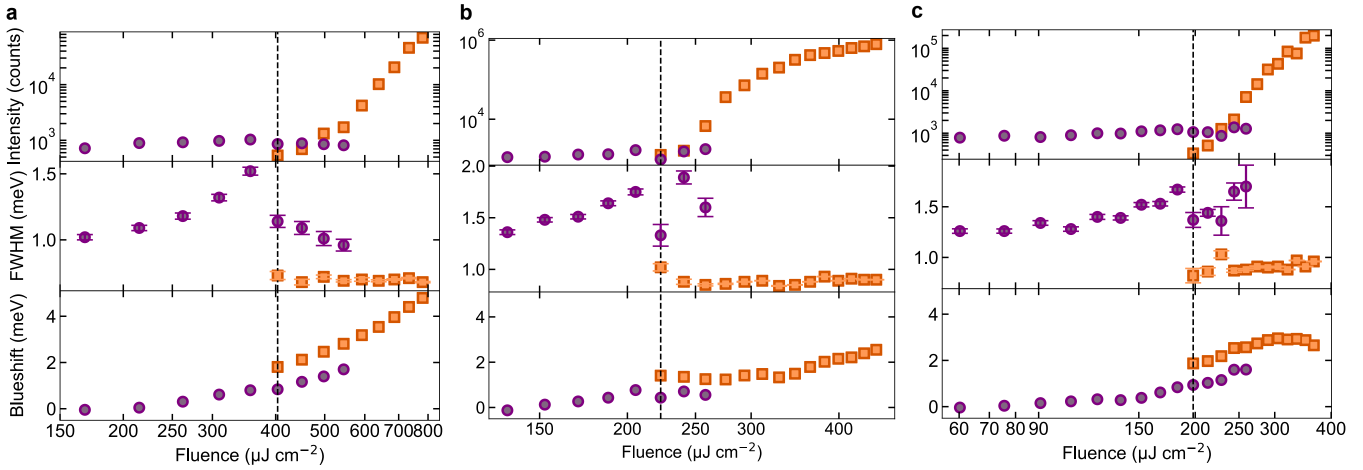

Supplementary Figure 9. **Additional condensation threshold measurements. a,** Additional threshold measurement at a different position of the main sample. **b-c,** Two extra measurements at different spots on a second, almost identical sample. The uncondensed LG00 polariton emission is denoted by purple discs and the LG00 condensate by orange squares. The panels show (top) emission intensity, (middle) emission linewidth, (bottom) emission peak energy. Error bars obtained from the fit errors are only significant for the uncondensed LG00 polariton emission linewidth. All three measurements show nonlinear increase of the emission and linewidth narrowing at threshold, and a blueshift of the condensate's emission. As mentioned in the main text, the threshold, linewidth and blueshift behavior can change between different spots, due to either material inhomogeneities and/or cavity variations (Q factor, longitudinal mode order etc.).

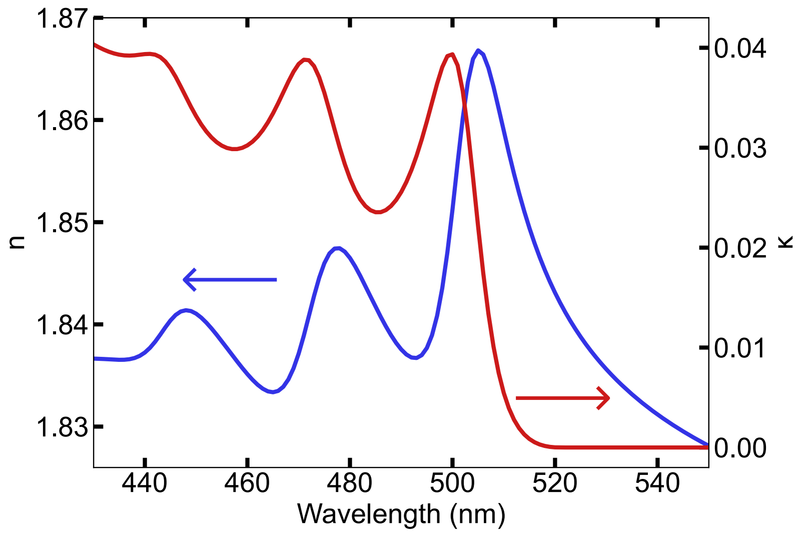

Supplementary Figure 10. **Real (n) and imaginary (κ) parts of the refractive index of a film of CsPbBr3 QDs with polystyrene, in blue and red respectively.** The presented measured components of the complex refractive index were used in the transfer matrix simulation presented in Supplementary Figure 8.
